# P-1647. Duration of SARS-CoV-2 Detection with Rapid Antigen Tests and Nucleic Acid Amplification Tests in a 2024 Nursing Home Resident Cohort

**DOI:** 10.1093/ofid/ofaf695.1822

**Published:** 2026-01-11

**Authors:** Katelin Jackson, Alfonso Hernandez, Majerle Reeves, Velma K Lopez, Tiffany Harris, Yasin Abul, David Canaday, Christopher J Crnich, Lindsay B LeClair, Samantha Fuller, Scott Fridkin, Jon P Furuno, Stefan Gravenstein, Steven Handler, Jennifer L Harcourt, Jessica Healy, Marc Lipsitch, Joseph D Lutgring, Jennifer K Meece, Alexandra Mellis, Lona Mody, David A Nace, Prabasaj Paul, Paulina Rebolledo, Hannah L Kirking, Rachel Slayton, Sujan Reddy, Morgan Katz

**Affiliations:** Centers for Disease Control and Prevention, Decatur, GA; CDC, Decatur, Georgia; CDC, Decatur, Georgia; Centers for Disease Control and Prevention, Decatur, GA; Abt Global, Rockville, Maryland; Brown University, Providence, Rhode Island; VA Northeast Ohio Healthcare System, Cleveland, OH; University of Wisconsin School of Medicine and Public Health, Madison, WI; Abt Global, Rockville, Maryland; Abt Global, Rockville, Maryland; Georgia Emerging Infections Program, Decatur, GA;Emory University School of Medicine, Atlanta, GA, Atlanta, Georgia; Oregon State University, Portland, Oregon; Brown University, Providence, Rhode Island; University of Pittsburgh, Pittsburgh, Pennsylvania; CDC, Decatur, Georgia; U.S. Centers for Disease Control and Prevention, Atlanta, Georgia; Harvard T.H. Chan School of Public Health, Boston, Massachusetts; Division of Healthcare Quality Promotion, Centers for Disease Control and Prevention, Atlanta, GA; Marshfield Clinic Research Institute, Marshfield, Wisconsin; Centers for Disease Control and Prevention, Decatur, GA; University of Michigan, Ann Arbor, Michigan; University of Pittsburgh, Pittsburgh, Pennsylvania; CDC, Decatur, Georgia; Emory University School of Medicine, Emory University Rollins School of Public Health, Atlanta, GA; Coronavirus and Other Respiratory Viruses Division, National Center for Immunization and Respiratory Diseases, CDC, Atlanta, GA; Centers for Disease Control and Prevention, Decatur, GA; CDC, Decatur, Georgia; Johns Hopkins, Stevenson, MD

## Abstract

**Background:**

Nursing home (NH) residents are at high risk of contracting and having adverse outcomes from SARS-CoV-2. Based on culture positivity CDC currently recommends 10-day transmission-based precautions; greater understanding of the duration of SARS-CoV-2 detection with rapid antigen tests (RAT) and nucleic acid amplification tests (NAAT), can further guide testing and isolation recommendations to reduce transmission. In a 2023 NH cohort with daily testing, the median duration of SARS-CoV-2 detection among NH residents was six days for RAT and 11 days for NAAT. We update estimates of SARS-CoV-2 detection duration in a 2024 cohort of NH residents to inform prevention efforts.

**Figure 1:**
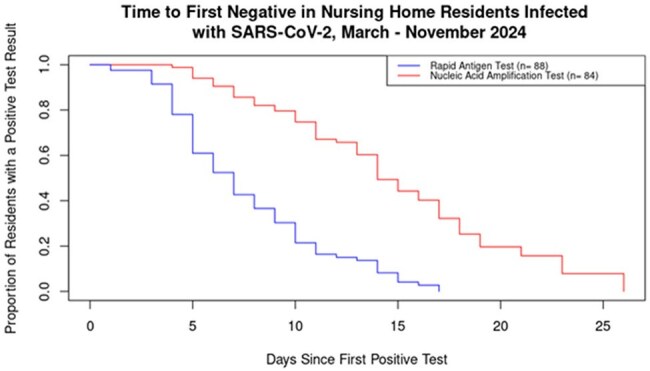
Kaplan-Meier analysis was restricted to residents with ≥4 RAT (n = 88) or NAAT (n = 84). A resident could be in both RAT and NAAT survival curves. The median duration of SARS-CoV-2 detection with RAT was seven days and the median duration of SARS-CoV-2 detection with NAAT was 14 days.

**Methods:**

We collected data on NH residents tested for SARS-CoV-2 with RAT and NAAT in 17 NHs across eight states. Residents could enroll upon a positive SARS-CoV-2 RAT or NAAT followed by paired nasal specimen collection every 3-5 days for up to 30 days. We abstracted resident clinical characteristics from available medical records. We used Kaplan-Meier (KM) survival analysis to estimate the time from first positive (day 0) to first negative. We restricted analyses to residents with ≥ 4 RAT or NAAT to capture the duration of SARS-CoV-2 positivity; residents could be in both RAT and NAAT survival curves. We used Greenwood’s formula to calculate 95% confidence intervals (CI).

**Results:**

We enrolled 121 residents with a positive SARS-CoV-2 test, of whom 87 (72%) were vaccinated. Eighty-five participants (70%) had a Charlson Comorbidity Index score ≥5. Sixty-eight participants received antivirals (n=36 [53%] molnupiravir; n=28 [41%] nirmatrelvir/ritonavir; n=4 [6%] remdesivir). The KM analysis included 88 (73%) and 84 (69%) residents with ≥ 4 RAT and NAAT, respectively. The median duration of SARS-CoV-2 detection with RAT was seven days; 61% and 21% had SARS-CoV-2 detection at five and 10 days, respectively (Figure 1). The median duration of SARS-CoV-2 detection with NAAT was 14 days; 94% and 75% had SARS-CoV-2 detection at five and 10 days, respectively.

**Conclusion:**

The median time from first positive to first negative test for NH residents with RAT was 7 days and 14 days for NAAT. The duration of SARS-CoV-2 detection among NH residents has not substantially changed since last season.

**Disclosures:**

Yasin Abul, MD, CDC/ABT: Grant/Research Support|CLARIO: Advisor/Consultant|GSK: Grant/Research Support|Moderna: Grant/Research Support|Seqirus: Grant/Research Support David Canaday, MD, Moderna: Grant/Research Support|Pfizer: Grant/Research Support|Seqirus: Advisor/Consultant|Seqirus: Grant/Research Support|Seqirus: Honoraria Christopher J. Crnich, MD, PhD, Merck: Grant/Research Support Samantha Fuller, MPH, AstraZeneca: Grant/Research Support|CSL Vifor: Grant/Research Support|GlaxoSmithKline: Grant/Research Support Jon P. Furuno, PhD, Merck: Grant/Research Support Stefan Gravenstein, MD, MPH, GSK: Advisor/Consultant|GSK: Grant/Research Support|GSK: Honoraria|Moderna: Grant/Research Support|Novavax: Advisor/Consultant|Novavax: Honoraria|Pfizer: Advisor/Consultant|Pfizer: Grant/Research Support|Pfizer: Honoraria|Sanofi: Advisor/Consultant|Sanofi: Grant/Research Support|Sanofi: Honoraria|Seqirus: Grant/Research Support Jennifer K. Meece, PhD, CSL Seqirus: Grant/Research Support|GSK: Grant/Research Support|ModernaTX: Grant/Research Support Lona Mody, MD, MS, Nanovibronix: Grant/Research Support|NIH, CDC, VA: Grant/Research Support|UpToDate: Honoraria Morgan Katz, MD, MHS, Skinclique: Advisor/Consultant

